# A Case of Paget-Schroetter Syndrome Successfully Treated With Endovascular Treatment

**DOI:** 10.7759/cureus.95666

**Published:** 2025-10-29

**Authors:** Tomohide Sato, Tomokazu Ikemoto, Ryusuke Tsunoda, Jyunjiro Koyama

**Affiliations:** 1 Division of Cardiology, Saiseikai Kumamoto Hospital Cardiovascular Center, Kumamoto, JPN; 2 Department of Cardiology, Japan Red Cross Kumamoto Hospital, Kumamoto, JPN

**Keywords:** catheter infusion, endovascular treatment (evt), paget-schroetter syndrome, urokinase, venous thoracic outlet syndrome

## Abstract

A 16-year-old male presented with a five-day history of numbness in the left upper limb. Physical examination revealed asymmetry in upper arm circumference: the circumference of the right upper arm was 25.5 cm, whereas that of the left was 28.5 cm. Contrast-enhanced computed tomography (CECT) revealed thrombotic occlusion of the left brachial vein, extending proximally to just before its confluence with the internal jugular vein. Evaluation of the thrombus at the elbow level was inconclusive due to poor contrast opacification. A diagnosis of Paget-Schroetter syndrome was made, and the patient was initiated on oral rivaroxaban 30 mg/day. Four days later, venous ultrasonography revealed no improvement, and the patient was admitted for further management. Endovascular therapy, including thrombus aspiration and balloon angioplasty, was performed, and an infusion catheter was placed for continuous administration of urokinase at 240,000 units/day. On day eight of hospitalization, venography confirmed satisfactory venous flow. No recurrence has been observed since then. Although the treatment strategies for Paget-Schroetter syndrome are diverse, this case demonstrates successful management with a combination of endovascular therapy and anticoagulation.

## Introduction

Paget-Schroetter syndrome (PSS), also referred to as effort thrombosis of the subclavian vein, is an extremely rare form of upper extremity deep vein thrombosis (DVT), with an estimated incidence of one to two cases per 100,000 population per year. Upper-extremity DVT accounts for approximately 1-4% of all DVT cases, and primary axillary thrombosis comprises only 10-20% of this already small proportion, making PSS an unusual clinical entity [1,2]. It predominantly affects men and typically presents in individuals in their 30s [3,4]. The underlying mechanism is thought to involve anatomical narrowing of the costoclavicular space, formed by the clavicle, first rib, anterior scalene muscle, and costoclavicular ligament, combined with repetitive upper-extremity motion. This results in chronic venous compression, endothelial injury, and subsequent thrombus formation in the subclavian vein [3,5].

Here, we report a case of PSS in a 16-year-old male, which was diagnosed early. Moreover, the prompt initiation of anticoagulation followed by endovascular therapy led to favorable outcomes.

## Case presentation

The patient was a 16-year-old male with no significant medical history, regular medication use, or relevant family history. He was a healthy second-year high school student and an active member of a competitive baseball team. The patient pitched to the right and batted to the left. Following an intensive practice session with a higher-than-usual training load, the patient developed numbness in his left upper limb upon returning home. He monitored his symptoms for several days; however, as they did not improve, he presented to our hospital six days after symptom onset.

**Figure 1:**
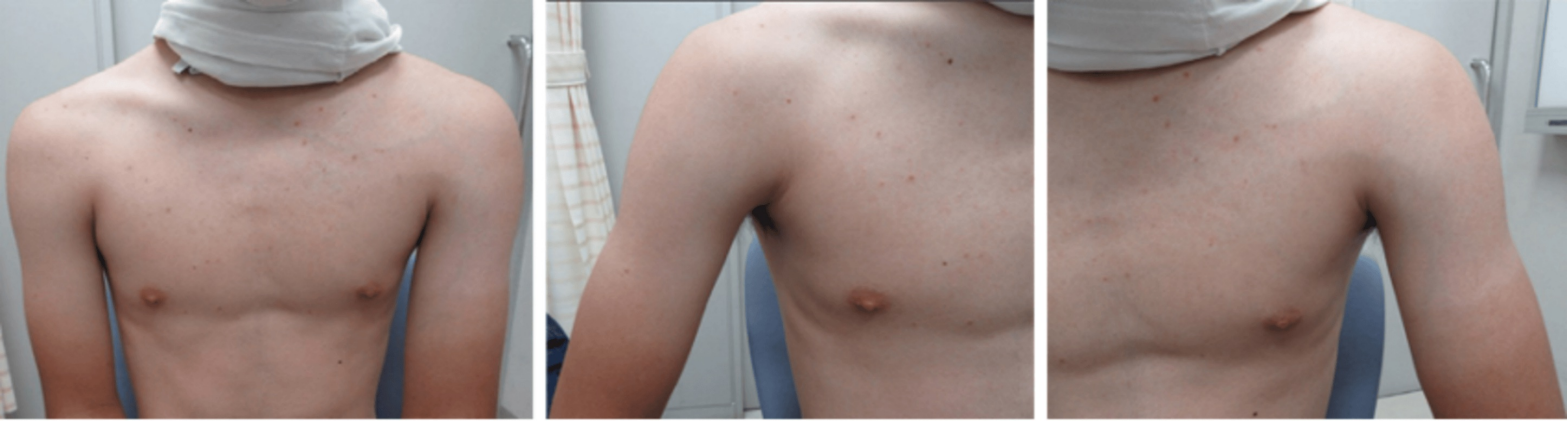
Clinical photograph of both upper limbs. Difference in upper arm circumference and dilated superficial veins observed on the left anterior chest wall.

On physical examination, his height and weight were 183 cm and 72.5 kg, respectively. An asymmetry was noted in upper arm circumference: 25.5 cm on the right and 28.5 cm on the left. Dilated superficial veins were observed over the left anterior chest wall (Figure 1). Tenderness was noted in the left axilla, but motor function was preserved.

The laboratory test results are listed in Table 1. D-dimer was mildly elevated at 1.4 μg/mL, and C-reactive protein (CRP) was also mildly elevated at 4.24 mg/dL, indicating an inflammatory response. Antinuclear antibody was negative; however, CH50 was ≥60.0 U/mL, lupus anticoagulant was 1.3, and anticardiolipin antibody was slightly elevated at 15.2 U/mL. Protein S activity was reduced by 61%.

**Table 1:** Laboratory findings on admission. D-dimer was mildly elevated at 1.4 μg/mL. C-reactive protein (CRP) was 4.24 mg/dL, indicating mild inflammation. Anticardiolipin antibody was slightly elevated (15.2 U/mL), and protein S activity was mildly reduced (61%).

Tests performed	Result	Reference range
White blood cells (U/mL)	7931	3100-8400
Red blood cells (×10^4^/μL)	481	431-556
Hemoglobin (g/dL)	14.2	14.0-17.5
Hematocrit (%)	41.8	40.2-50.8
Platelet count (×10^4^/μL)	26.4	15.6-37.3
Total protein (g/dL)	7.6	6.5-8.5
Albumin (g/dL)	4.6	4.0-5.2
Sodium (mEq/L)	137	135-145
Potassium (mEq/L)	4.3	3.5-5.0
Chloride (mEq/L)	106	96-107
Blood urea nitrogen (mg/dL)	11.2	9-21
Creatinine (mg/dL)	0.82	0.5-0.8
Glucose (mg/dL)	97	65-109
Glutamic-oxaloacetic transaminase (IU/L)	23	5-37
Glutamic-pyruvic transaminase (IU/L)	15	6-43
Lactate dehydrogenase (IU/L)	250	124-222
Total cholesterol (mg/dL)	138	150-219
Triglycerides (mg/dL)	32	30-149
C-reactive protein (mg/dL)	4.24	<0.30
B-type natriuretic peptide (pg/mL)	2.8	<18.4
Glycated hemoglobin (%)	5.6	4.6-6.2
Thyroid-stimulating hormone (μgIU/mL)	0.825	0.5-5.00
Free triiodothyronine (pg/mL)	3.12	2.4-4.5
Free thyroxine (ng/dL)	1.08	1.0-1.7
Activated partial thromboplastin time (seconds)	27.2	25.0-40.0
Prothrombin time	1.04	0.9-1.1
D-dimer (μg/mL)	1.4	<1.0
Antithrombin III	100	80-130
Ferritin (ng/mL)	131.5	13-301
Protein C (%)	69	64-146
Protein S (%)	61	60-150
Total hemolytic complement (U/mL)	>60	30-40
Rheumatoid factor	<3	Negative
Anti-nuclear antibody	<40	<40
Anti-Sm antibody	Negative	Negative
Lupus anticoagulant	1.3	<1.3
Anti-cardiolipin antibody (UA/mL)	15.2	＜10
Anti-double-stranded deoxyribonucleic acid antibody (U/mL)	<10	<10
β2 glyco-protein 1 dependent anti-cardiolipin (U/mL)	<1.2	<3.5

**Figure 2:**
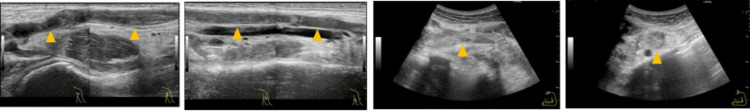
A venous ultrasound of the left upper extremity. Venous ultrasound of the left upper extremity showing thrombus formation from the subclavian vein to the mid-brachial vein (yellow arrows).

A venous ultrasound of the left upper extremity is shown in Figure 2. Thrombus formation was observed from the subclavian vein to the mid-brachial vein (yellow arrows). No Doppler flow signals were detected, indicating near-complete occlusion. The thrombus showed high echogenicity, suggesting a subacute-to-chronic stage. No thrombi were detected in the veins distal to the elbow.

**Figure 3:**
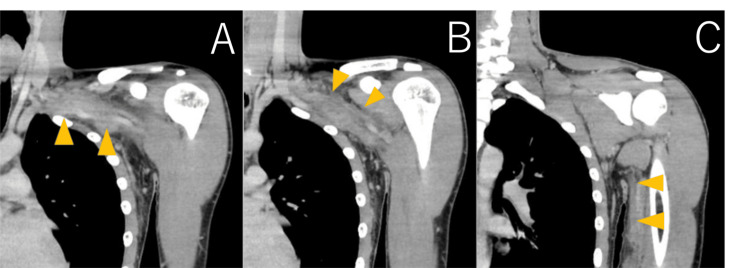
The contrast-enhanced computed tomography (CECT) images (A-C). CECT showing thrombotic occlusion of the left brachial vein extending up to the confluence with the internal jugular vein.

The contrast-enhanced computed tomography (CECT) images are shown in Figure 3. The left brachial vein demonstrated thrombotic occlusion extending up to the confluence with the internal jugular vein. Assessment of the thrombus at the elbow level was inconclusive because of poor opacification caused by the existing thrombus.

The diagnosis of primary subclavian vein thrombosis or PSS was established based on three key findings: (1) symptom onset following excessive activity, (2) radiologic evidence of subclavian vein thrombosis with pain and swelling of the left upper extremity, and (3) the absence of identifiable secondary causes. Following the diagnosis, initial management included oral rivaroxaban 30 mg/day. However, repeat venous ultrasonography performed four days later revealed a persistent thrombosis without improvement, and the patient was admitted for endovascular therapy.

**Figure 4:**
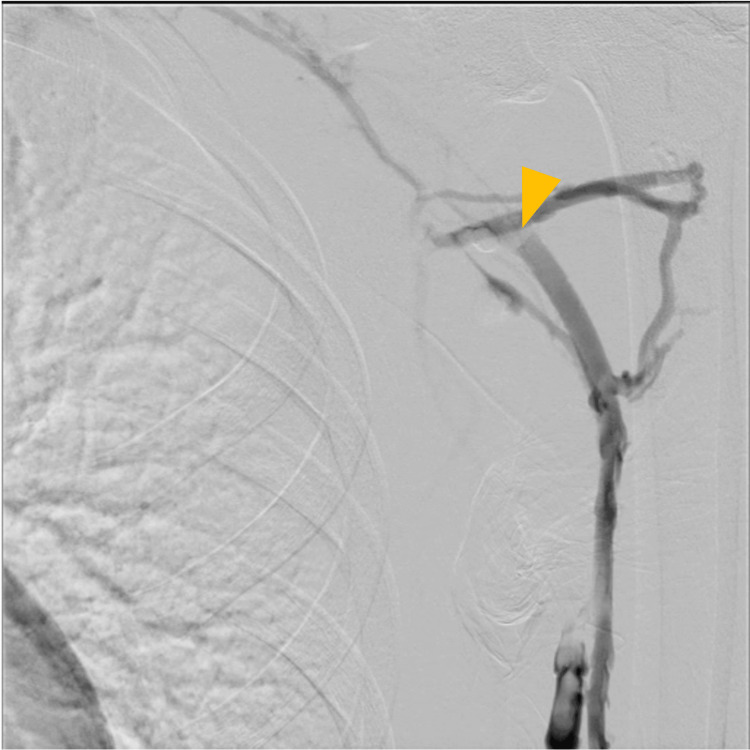
Endovascular therapy (day 1). Extensive thrombus observed from the subclavian vein to the brachial vein (yellow arrows).

**Figure 5:**
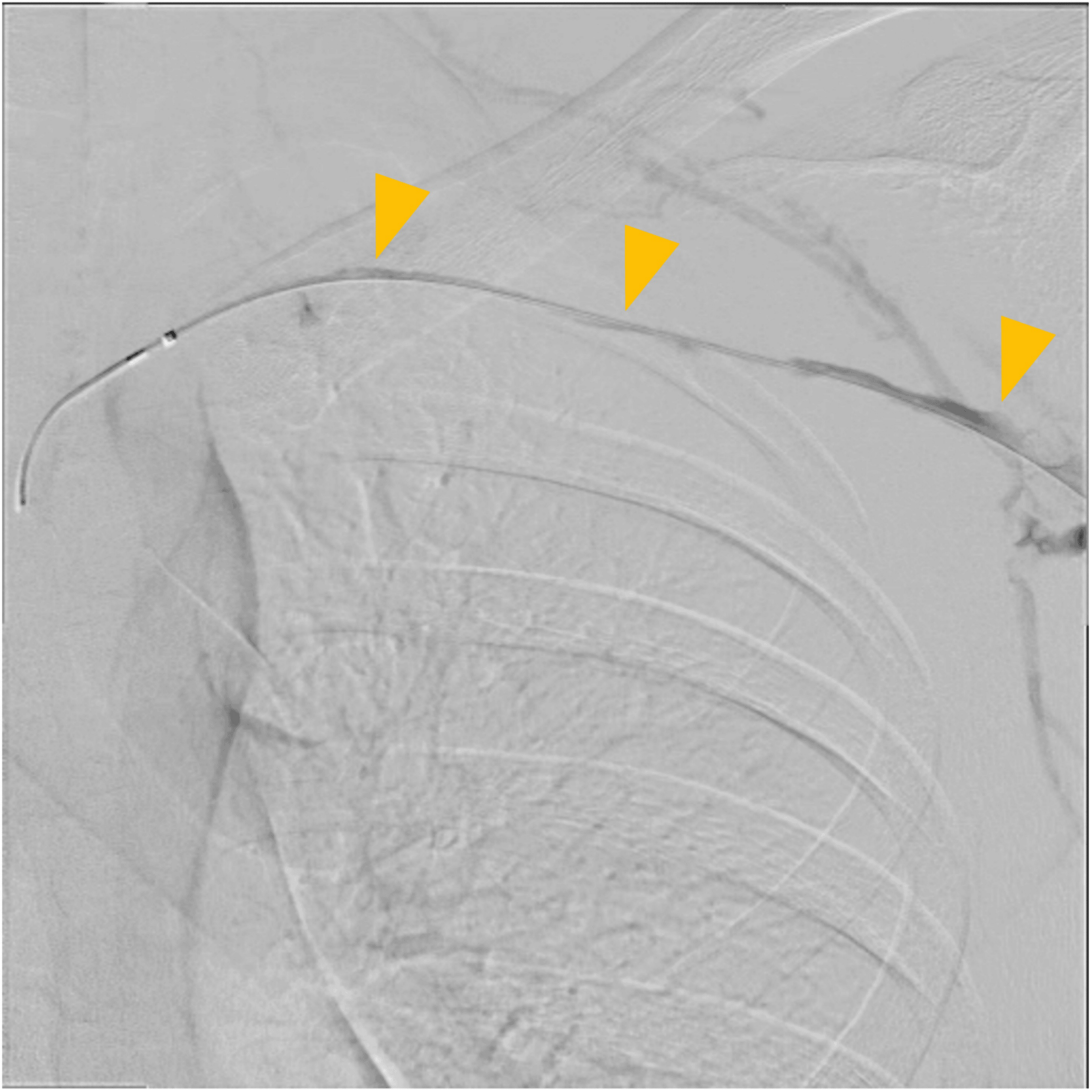
Endovascular therapy (day 1). Final venography after treatment demonstrating venous flow into the superior vena cava (yellow arrows).

Following admission, an 8 Fr sheath was inserted via the left cubital vein. Venography revealed an extensive thrombus extending from the subclavian vein to the brachial vein (Figure 4). Thrombus aspiration was performed using an 8 Fr manual aspiration catheter, followed by balloon angioplasty with a 4.0 × 100 mm balloon. A 4.0 × 100 mm balloon was initially selected based on venographic estimation of vessel caliber and to minimize the risk of vessel injury during the first dilation. Final venography confirmed restoration of venous flow into the superior vena cava (Figure 5). An infusion catheter was placed, and continuous urokinase infusion was initiated at 240,000 units/day.

**Figure 6:**
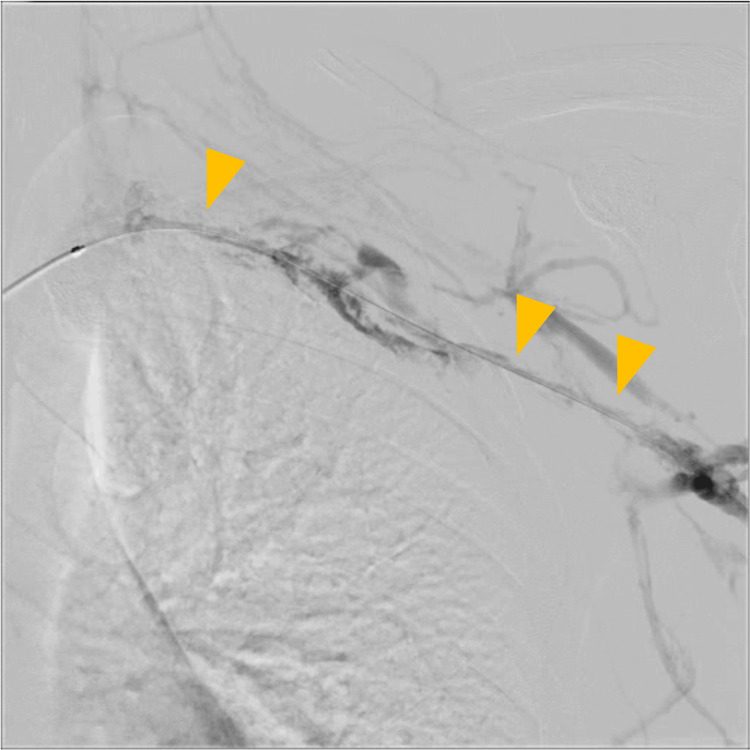
Endovascular therapy (day 5). Venography on hospital day 5, showing poor venous flow (yellow arrows).

**Figure 7:**
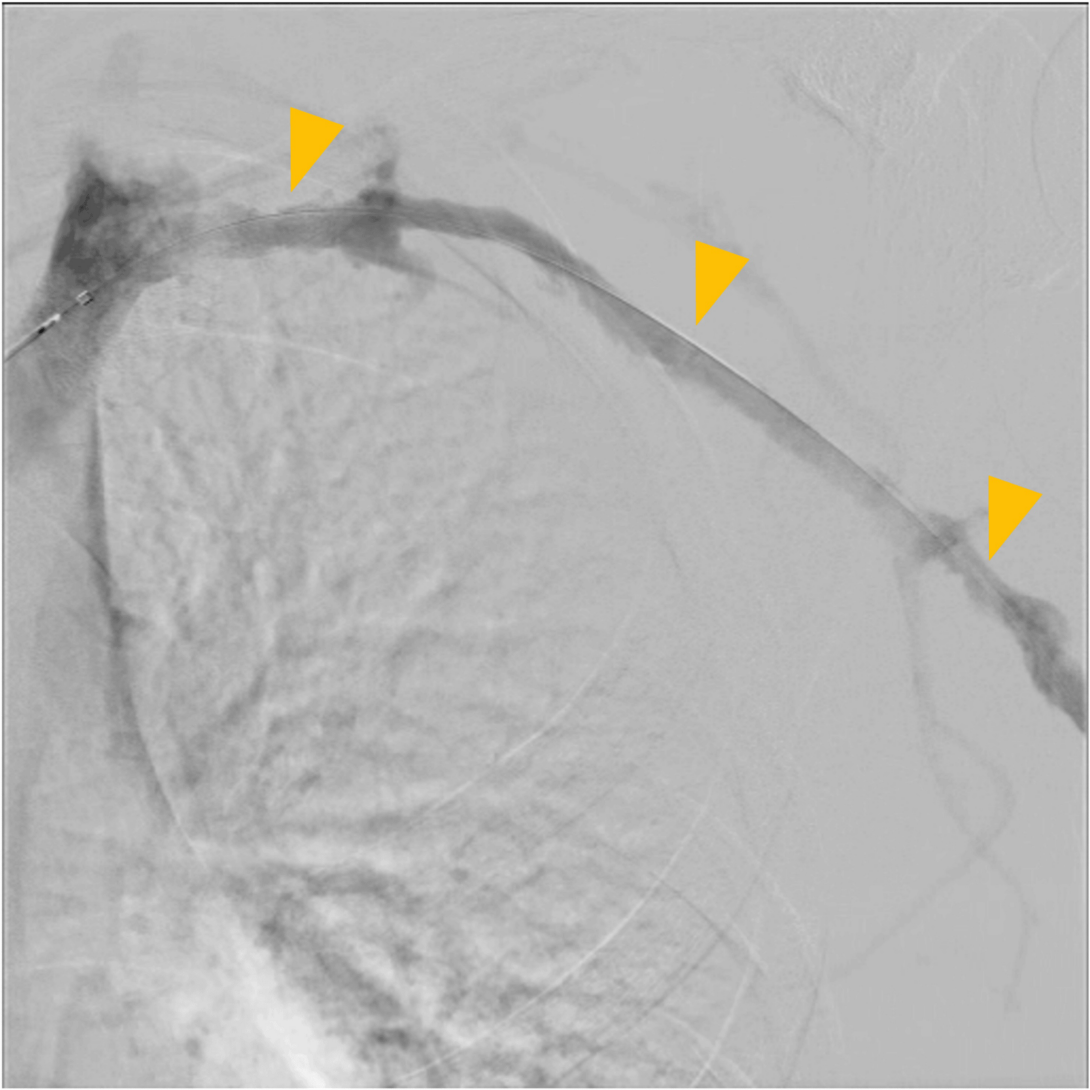
Endovascular therapy (day 5). Final venography after treatment showing increased venous flow into the superior vena cava compared with the previous procedure (yellow arrows).

On hospital day 5, follow-up venography revealed suboptimal venous flow (Figure 6). Therefore, endovascular therapy was repeated on the same day. This time, access was obtained via a different venous route, from the axillary to the brachial vein. Thrombus aspiration was again performed with an 8 Fr aspiration catheter, and the stenotic site was dilated with a 5.0 × 20 mm cutting balloon followed by a 7.0 × 100 mm balloon. Final venography demonstrated improved flow into the superior vena cava compared with the initial procedure (Figure 7). The infusion catheter was reinserted, and urokinase infusion was continued at the same dosage.

Follow-up venography was performed on hospital day 5 to evaluate residual thrombus and flow restoration. Because venous flow remained suboptimal, repeat thrombolysis was performed via a different venous access (axillary-to-brachial approach) to improve catheter positioning and drug delivery. Thrombolysis was continued until satisfactory venous flow was confirmed on day 8, and the treatment endpoint was determined based on both venographic appearance and clinical improvement.

**Figure 8:**
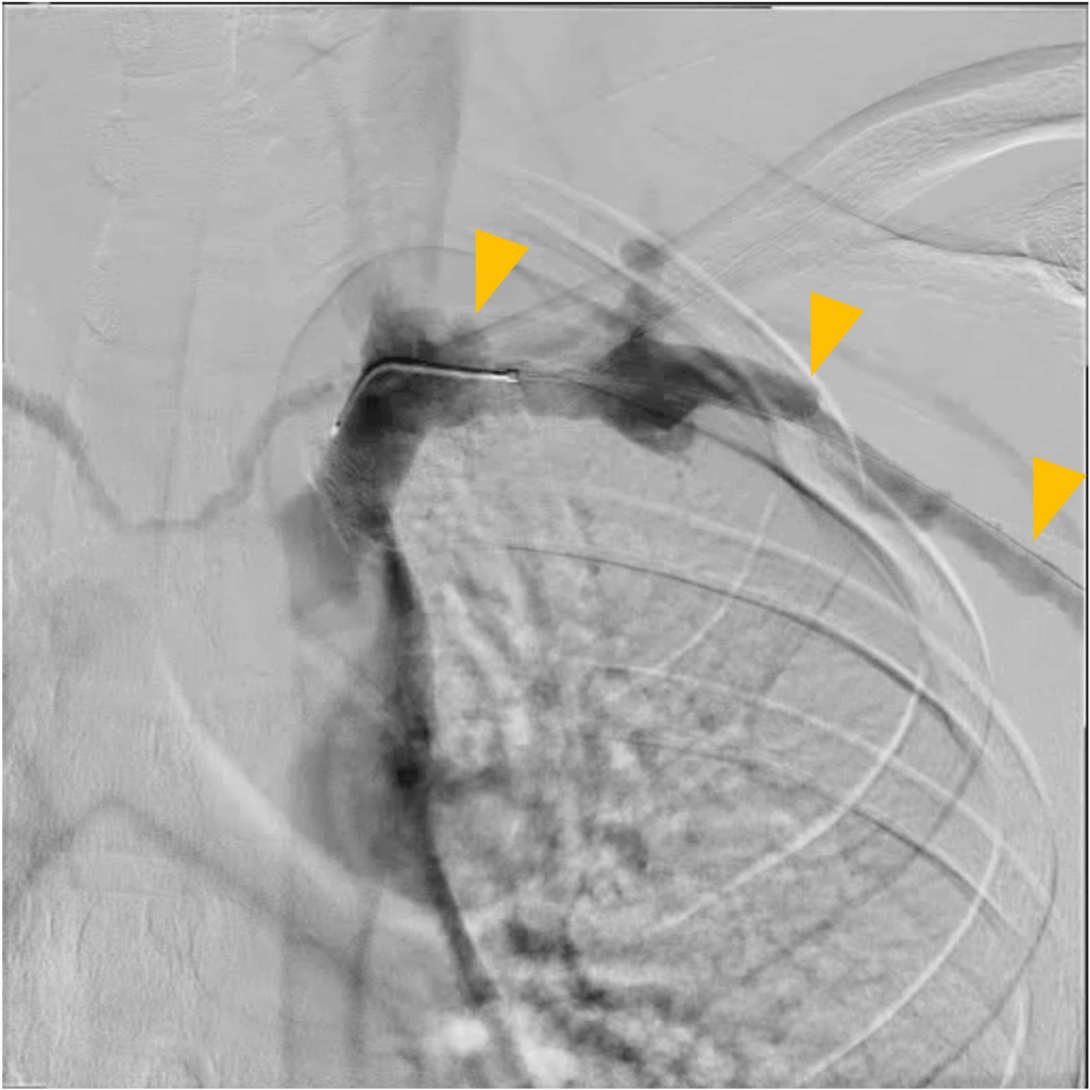
Endovascular therapy (day 8). Venography on hospital day 8, demonstrating sustained satisfactory venous flow (yellow arrows).

On hospital day 8, venography confirmed satisfactory venous flow (Figure 8), and the patient was discharged.

After discharge, the patient’s initial symptoms of numbness and pain resolved. Contrast-enhanced CT performed two weeks later revealed patchy ground-glass opacities in the lung fields, raising the possibility of pulmonary embolism. However, there were no clinical findings such as dyspnea or oxygen desaturation. Therefore, observation was opted for. On mediastinal window images, a residual thrombus within the left axillary to subclavian vein was suspected, but this had decreased in size compared with the previous study. No echocardiographic abnormalities were noted; cardiac chamber dimensions and function were within normal limits, and no thrombus extension into the superior vena cava, right atrium, or pulmonary artery was observed. Two months after discharge, upper limb ultrasonography demonstrated a further reduction in thrombus size, indicating improvement. The patient continued to have regular outpatient follow-up with no evidence of re-occlusion or recurrent stenosis. 

## Discussion

PSS is considered an extremely rare condition with a reported prevalence of one to two cases per 100,000 individuals and predominantly affects men in their 30s [3,4,6,7]. It is thought to result from the anatomical narrowing of the costoclavicular space, which is formed by the clavicle, first rib, anterior scalene muscle, and costoclavicular ligament, together with repetitive upper-limb activity. These factors contribute to chronic venous compression, intimal injury, and thrombus formation in subclavian veins. Excessive physical exertion, such as that associated with sports, is often a precipitating factor, as observed in the present case [5].

Treatment strategies for PSS include anticoagulation, percutaneous angioplasty, stent placement, surgical decompression, and patch venoplasty. These approaches may be used in combination depending on the clinical situation. However, due to the rarity of this condition, no standardized treatment guidelines exist [8-11]. In the present case, the combination of anticoagulation and percutaneous angioplasty was effective, and no recurrence has been observed to date. Nevertheless, anticoagulation therapy alone is associated with a high rate of recurrence, even when initiated early [7,11], and percutaneous angioplasty is associated with poor long-term patency [11].

Surgical intervention is considered the most definitive therapy. First-rib resection and other thoracic outlet decompression procedures have been reported to provide favorable long-term outcomes [9]. Furthermore, the postoperative quality of life is not significantly limited by such procedures. Thompson et al. also demonstrated the efficacy of paraclavicular venolysis and decompression [8]. However, in this particular case, surgery requiring a period of postoperative rest was avoided because the onset occurred just before an important competitive match. There is an ongoing debate regarding the necessity of surgical decompression after successful thrombolysis. While some authors advocate for first-rib resection to prevent recurrence, others report favorable outcomes with endovascular management alone when no fixed anatomical compression is demonstrated [3]. In our case, given the absence of fixed obstruction on intravascular ultrasonography (IVUS) and the patient’s competitive athletic schedule, surgery was deferred with close follow-up.

One reason for the success of endovascular therapy in this case may be the selection of an alternative venous access route for the second intervention. In cases of venous occlusion due to a thrombus, it is extremely difficult to identify vessels with sufficient caliber and flow to achieve effective results. If the initial intervention is inadequate, additional treatment via an alternative venous route should be considered.

With regard to the mechanism of occlusion in this patient, chronic obstruction may have led to venous degeneration or fixed anatomical narrowing. Therefore, IVUS was performed, but no venous degeneration or fixed obstruction was identified; only a thrombus adhering to the venous wall was observed. No anatomical abnormalities such as first-rib anomalies, anterior scalene muscle hypertrophy, or costoclavicular ligament thickening were observed on imaging, suggesting that functional rather than structural compression was responsible. Recent studies have shown that IVUS can detect subclavian vein stenosis more sensitively than venography, particularly in cases without obvious compression on conventional imaging [12].

Although minimizing repetitive upper-extremity stress is generally recommended, this was difficult in our case because the patient was a competitive baseball player aspiring to a professional career. If the patient continues to pursue professional athletic performance, ongoing anticoagulation may be necessary, given the training demands. Conversely, if he pursues a nonathletic career, upper limb stress is expected to be considerably lower, and long-term anticoagulation may not be required. Regarding the need for surgery in the future, if recurrence occurs after discontinuation of anticoagulation therapy, initial management should prioritize anticoagulation and endovascular therapy, with surgery considered based on the results. Given that the IVUS revealed no venous degeneration or fixed obstruction, continued oral anticoagulation appears to be the most reasonable management at present. However, shared decision-making with the patient remains essential.

## Conclusions

We report a case of PSS that developed following vigorous physical activity and achieved favorable clinical outcomes. In young patients presenting with upper-extremity numbness or swelling, prompt recognition and management are essential, and PSS should be considered in the differential diagnosis. In the differential diagnosis of upper-extremity swelling and pain, thoracic outlet syndrome (TOS) should also be considered, and physical maneuvers such as the Adson, Wright, and Roos tests may be useful for bedside screening. It is also important to comprehensively evaluate the treatment options, taking into account the patient’s background and clinical context.
